# Supplementary material for: Cofactors of earlier uptake of modern postpartum family planning methods in Kenya
Source: PLOS Glob Public Health. 2025 Jun 6;5(6):e0004353. doi: 10.1371/journal.pgph.0004353 (PMC12143578; doi:10.1371/journal.pgph.0004353)
Supplement: Attachment — Submitted filename: Reviewers comments.docx [file pgph.0004353.s002.docx]

Suggested queries and improvements that could enhance the research:

1. Why does the author not include an analysis of the socio-economic advantages of contraceptive uptake among women in the postpartum period, considering how these factors may influence access to and utilization of family planning services?
2. Could you consider introducing a paragraph that outlines the history of programs and policies related to reproductive health in Kenya since independence? This context could provide valuable insights into the evolution of family planning services and their integration into postnatal care, enriching the understanding of current challenges and opportunities in postpartum family planning uptake.
3. One potential gap in the methodology is the exclusion of women who did not attend any postnatal follow-up visits. This could introduce selection bias, as those who attend follow-up visits may differ significantly from those who do not, potentially affecting the generalizability of the findings. Additionally, excluding women who experienced miscarriages, stillbirths, or molar pregnancies may limit the understanding of the experiences and needs of all women facing adverse pregnancy outcomes, which could provide valuable insights into the overall postpartum care landscape and its impact on family planning uptake. Including a broader range of experiences could enrich the data and inform more inclusive health policies.
4. Other family planning methods that could be considered but are excluded from the defined modern methods include:

- Traditional Methods
- Periodic Abstinence: Avoiding sexual intercourse during fertile periods (e.g., calendar method, rhythm method).
- Withdrawal: The practice of pulling out before ejaculation.
- Lactational Amenorrhea Method (LAM)**: Using breastfeeding as a temporary method of contraception.
- Natural Family Planning: Techniques that track fertility signs (e.g., basal body temperature, cervical mucus monitoring)
- Emergency Contraception: Pills taken after unprotected intercourse to prevent pregnancy (e.g., Plan B, Ella).
- Spermicides: Chemical products that kill sperm, often used with barrier methods.

Including these methods could provide a more comprehensive understanding of family planning options available to women, particularly in different cultural contexts.

5. Based on the findings regarding later uptake of postpartum family planning (FP) among specific groups of women, the following policy recommendations can be made:

- Targeted Education and Outreach: Develop tailored educational programs aimed at adolescent girls and women with lower levels of education. These programs should focus on the importance of postpartum FP and provide information on available methods, addressing myths and misconceptions.
- Strengthening Partner Involvement: Implement initiatives that encourage partner involvement in family planning discussions and decisions. This could include counseling sessions that involve both partners, aiming to promote shared responsibility for contraceptive use.
- Enhancing Social Support Networks: Create community-based support systems that provide social and emotional support to postpartum women, particularly those without partners. Peer support groups can help reduce feelings of isolation and improve access to information about FP options.
- Financial Assistance Programs: Establish financial support mechanisms for women who lack financial resources from their partners. This could involve subsidies for contraceptive methods or support for accessing healthcare services.
- Special Programs for Primigravid Women: Develop specific programs for first-time mothers that address their unique concerns and needs regarding postpartum FP, including counseling on coping with new motherhood and family planning options.
- Addressing History of Adverse Pregnancy Outcomes: Provide targeted counseling and support for women with a history of miscarriages or stillbirths, helping them to understand their reproductive health and the benefits of FP.
- Integration of FP Services into Maternal Health Care: Ensure that family planning services are integrated into routine maternal and child health visits, particularly for women with young children, to facilitate discussions about postpartum FP.
- Ambivalence and Counseling: Offer specialized counseling for women who express ambivalence about their recent pregnancy, helping them to explore their feelings and understand their options for family planning.

Thank you

Reviewer
