# Supplementary material for: Cofactors of earlier uptake of modern postpartum family planning methods in Kenya
Source: PLOS Glob Public Health. 2025 Jun 6;5(6):e0004353. doi: 10.1371/journal.pgph.0004353 (PMC12143578; doi:10.1371/journal.pgph.0004353)
Supplement: Attachment — Submitted filename: Response letter to reviewers_10122024.docx [file pgph.0004353.s004.docx]

**KENYATTA NATIONAL HOSPITAL**	Tel.: 2726300/2726450/2726565

P.O. Box 20723-00202 Nairobi	Research & Programs: Ext. 44705

Fax: 2725272

Email: [knhresearch@gmail.com](mailto:knhresearch@gmail.com)

Date: 11^th^ December 2024

Dear Editor,

We are grateful for the comments on the submission of our manuscript, “*Cofactors of earlier uptake of modern postpartum family planning methods in Kenya”*

We have addressed the comments raised below in italic.

We appreciate your consideration of this manuscript for publication in the PLOS Global Public Health journal. Please contact me if you have any questions regarding this resubmission.

Sincerely,

Author

Comments

1. We note that you have indicated that data from this study are available upon request. As you may be aware, PLOS journals require authors to make all data underlying their findings fully available without restrictions at the time of publication (<https://journals.plos.org/globalpublichealth/s/data-availability>). If specific legal or ethical constraints prevent public sharing of a dataset, authors must explain how others can access the data.

*Thank you for this comment. As advised, we have uploaded the minimal anonymized dataset necessary to replicate the study findings to a stable, public repository. The data that support the findings of this study are publicly available on GitHub - https://github.com/Ngumbau-Nancy/Cofactors-of-FP-uptake”*
