# Supplementary material for: Cofactors of earlier uptake of modern postpartum family planning methods in Kenya
Source: PLOS Glob Public Health. 2025 Jun 6;5(6):e0004353. doi: 10.1371/journal.pgph.0004353 (PMC12143578; doi:10.1371/journal.pgph.0004353)
Supplement: Attachment — Submitted filename: Response to reviewers_06012025.docx [file pgph.0004353.s005.docx]

**KENYATTA NATIONAL HOSPITAL**	Tel.: 2726300/2726450/2726565

P.O. Box 20723-00202 Nairobi	Research & Programs: Ext. 44705

Fax: 2725272

Email: [knhresearch@gmail.com](mailto:knhresearch@gmail.com)

Date: 6^th^ January 2025

Dear Editor,

We are grateful for the thoughtful comments on our manuscript, “*Cofactors of earlier uptake of modern postpartum family planning methods in Kenya”*

We have addressed the comments provided by the editorial office and the reviewers’ point by point below in italic, and have revised our manuscript and figures.

We appreciate your consideration of this manuscript for publication in the PLOS Global Public Health journal. Please contact me if you have any questions regarding this resubmission.

Sincerely,

Author

Editorial office comments

Please send a completed 'Competing Interests' statement, including any COIs declared by your co-authors. If you have no competing interests to declare, please state "The authors have declared that no competing interests exist". Otherwise please declare all competing interests beginning with the statement "I have read the journal's policy and the authors of this manuscript have the following competing interests:"

*Thank you for this comment. As advised, we have adjusted our ‘Competing Interest’ statement in the manuscript and the portal to read as follows:*

*“The authors have declared that no competing interests exist. JMB is employed by Gilead Sciences, outside of this work.”*

2. Please amend your detailed Financial Disclosure statement. This is published with the article. It must therefore be completed in full sentences and contain the exact wording you wish to be published.

i. State the initials, alongside each funding source, of each author to receive each grant. For example: ""This work was supported by the National Institutes of Health (####### to AM; ###### to CJ) and the National Science Foundation (###### to AM)."

ii. State what role the funders took in the study. If the funders had no role in your study, please state: “The funders had no role in study design, data collection and analysis, decision to publish, or preparation of the manuscript.”

*Thank you for this comment. We have amended our Financial Disclosure statement in the manuscript and the portal to read as follows:*

*“This study was funded through the National Institute of Allergy and Infectious Disease grant (R01 AI25498 to GJS) and Eunice Kennedy Shriver National Institute of Child Health & Human Development (R01 HD094630, R01HD100201 to GJS). The funders had no role in study design, data collection and analysis, decision to publish, or preparation of the manuscript.”*

We note that you have indicated that data from this study are available upon request. As you may be aware, PLOS journals require authors to make all data underlying their findings fully available without restrictions at the time of publication (<https://journals.plos.org/globalpublichealth/s/data-availability>). If specific legal or ethical constraints prevent public sharing of a dataset, authors must explain how others can access the data.

*Thank you for this comment. As advised, we have uploaded the minimal anonymized dataset necessary to replicate the study findings to a stable, public repository. The data that support the findings of this study are publicly available on GitHub -* [*https://github.com/Ngumbau-Nancy/Cofactors-of-FP-uptake*](https://github.com/Ngumbau-Nancy/Cofactors-of-FP-uptake)*”*

Reviewer’s Comments

REVIEWER 1: This is an important topic and a well conducted study. Suggested edits:

Background:

Abstract: Consider adding ‘in high HIV prevalence settings’ for accuracy and representation of your study population. For example: “There are limited data on uptake of postpartum family planning (FP) in resource-limited settings, particularly in high HIV prevalence settings. We assessed the timing of postpartum FP initiation and the cofactors of earlier uptake of modern postpartum FP in Kenya”

*Thank you for this comment. As advised, we have added ‘in high HIV prevalence settings’ in the background* *for accuracy and representation of my study population. This has also been updated in the study population section of the manuscript now reading:*

*“There are limited data on uptake of postpartum family planning (FP) in resource-limited settings, particularly in high HIV prevalence settings”*

*This study utilized longitudinal data from the ‘PrEP Implementation of Mothers in Antenatal care’ (PrIMA) study, a cluster-randomized clinical trial that evaluated two models of pre-exposure prophylaxis (PrEP) delivery among pregnant women living in high HIV prevalence settings (risk-based versus universal delivery) – (NCT03070600)*

For precision, consider highlighting the contribution of your study to the research. For example: Resumption of sex occurred earlier than uptake of postpartum FP. Our findings underscore the importance of addressing individual, interpersonal, and obstetric factors to tailor postpartum FP counseling and interventions, particularly in high HIV prevalence settings.

*Thank you for this comment. We have highlighted the contribution of this study as follows:*

*“Resumption of sex occurred earlier than uptake of postpartum FP. Our findings underscore the importance of addressing the individual, interpersonal, social and obstetric factors associated with timeliness of postpartum FP uptake. In development of interventions to improve uptake of postpartum FP, these factors should be considered, particularly in high HIV prevalence settings.”*

The introduction jumps between global and Kenya-specific statistics. Reorganize to present a clearer narrative and improve the flow:

a. Global burden of unmet need for FP, with a focus on sub-Saharan Africa.

b. FP situation in Kenya (national and regional variations).

c. Importance of postpartum FP and the knowledge gaps this study addresses.

*We have edited the manuscript to include a paragraph on the global burden of unmet need for FP with a focus on sub-Saharan Africa (paragraph 1) and the FP situation in Kenya; national and regional variations (paragraph 2).*

*We have also edited the manuscript to include the importance of postpartum FP and the knowledge gaps that this study addresses (paragraph 3 and 4)*

Methods: Though it is mentioned, consider clearly stating that this is a secondary analysis of data from a larger study in your study design.

*Thank you for this comment. We have clearly stated that this is a secondary analysis of data from a larger study as follows:*

*“This study is a secondary data analysis of longitudinal data collected for the ‘PrEP Implementation of Mothers in Antenatal care’ (PrIMA) study, a cluster-randomized clinical trial that evaluated two models of pre-exposure prophylaxis (PrEP) delivery among pregnant women living in high HIV prevalence settings (risk-based versus universal delivery) – (NCT03070600)”*

While the definition of ‘Modern FP’ is provided, consider adding a brief explanation of why this distinction is important (e.g., effectiveness, user dependence).

*Thank you for this comment. We have added an explanation of what distinguishes our definition of modern FP from the rest as follows:*

*“We defined modern FP methods as products or medical interventions that interfere with reproduction allowing women to have sexual intercourse at any desired time. These methods include tubal ligation, vasectomy, intrauterine contraceptive device (IUD), implants, injectables (Depo provera), oral contraceptive pills, and condoms (male or female)”*

Acknowledge that a validated scale was not used to determine ‘Pregnancy Intention’ and explain the rationale for the chosen method, as mentioned in the ‘Discussion’ section.

*Thank you for this comment. We have acknowledged that a validated scale was not used to determine ‘pregnancy intention’ and the rationale as follows:*

*“Since the parent study did not include a validated scale to measure pregnancy intention, pregnancies were characterized as intended, unintended or ambivalent based on questions that asked participants who had delivered what they felt about the last pregnancy.”*

In your data analysis, specify the time origin for the survival analysis (date of delivery). Also, briefly describe the process of selecting covariates for the Cox models.

*We have clarified the time of origin for the survival analysis in the data analysis section as the time that the participant delivered to the earliest visit with a record of using postpartum FP on or before the 9-month visit. We have also clarified the process of selecting covariates for the Cox models as follows:*

*“Potential cofactors for the cox proportional hazard models, including sociodemographic characteristics, pregnancy characteristics, and partnership dynamics, were selected a priori based on associations with uptake of postpartum FP noted in previous literature, or the research team’s intuition informed by their clinical practice”*

Results: Consider a more informative caption for figure 1, such as "Cumulative Incidence of Sexual Resumption and Postpartum FP Uptake."

*Thank you for this comment. We have edited the caption for figure 1 to read:*

*“Cumulative incidence of sexual resumption and postpartum FP uptake in the overall population”*

I wonder if a forest plot would complement figure 3 to present hazard ratios and confidence intervals more effectively.

*Thank you for this comment. We have included forest plots to present the hazard ratios and confidence intervals more effectively.*

Discussion: Good summary of key findings; Good association with existing literature; exhaustive strengths and limitations (large sample size, prospective design, and use of appropriate statistical methods). Consider adding a specific and actionable recommendation for interventions and policies (Is it generalizable? Consider HIV +ve population) such as counseling on the risks of pregnancy during the postpartum period and the benefits of long-acting reversible contraceptives (Or peer support groups, financial incentives, and male involvement programs).

*Thank you for this comment. We have added actionable recommendations for interventions and policies in the discussion section.*

Owing to the study’s significance, a call for further research to understand the reasons behind the observed associations is appropriate.

*Thank you for this comment. We have included a call for further research as follows:*

*“Further research is needed on different strategies for delivery of FP to postpartum women to overcome delays in FP initiation, taking into account their individual, interpersonal, and obstetric needs and perspectives.”*

REVIEWER 2:

Why does the author not include an analysis of the socio-economic advantages of contraceptive uptake among women in the postpartum period, considering how these factors may influence access to and utilization of family planning services?

*Thank you for this comment. An analysis of the socio-economic advantages of contraceptive uptake among women in the postpartum period has been included in paragraph 3*

Could you consider introducing a paragraph that outlines the history of programs and policies related to reproductive health in Kenya since independence? This context could provide valuable insights into the evolution of family planning services and their integration into postnatal care, enriching the understanding of current challenges and opportunities in postpartum family planning uptake.

*Thank you for this comment. We have outlined the history of programs and policies related to contraceptive uptake in Kenya since 1967 in paragraph 3*

One potential gap in the methodology is the exclusion of women who did not attend any postnatal follow-up visits. This could introduce selection bias, as those who attend follow-up visits may differ significantly from those who do not, potentially affecting the generalizability of the findings. Additionally, excluding women who experienced miscarriages, stillbirths, or molar pregnancies may limit the understanding of the experiences and needs of all women facing adverse pregnancy outcomes, which could provide valuable insights into the overall postpartum care landscape and its impact on family planning uptake. Including a broader range of experiences could enrich the data and inform more inclusive health policies.

*Thank you for this comment. We have added the highlighted gaps in the methodology in the study limitations section of the manuscript as follows:*

*“Although the study visits mirrored the required postnatal and infant immunization visits, we excluded women who did not attend any postnatal follow-up visits to determine the median time to postpartum resumption of sexual activities and uptake of modern postpartum FP method. By doing so, we may have introduced selection bias by capturing women who have higher health-seeking behaviors potentially affecting the generalizability of the findings. Additionally, excluding women who experienced miscarriages, stillbirths, or molar pregnancies may limit the understanding of the needs and impact of adverse pregnancy outcomes on uptake of postpartum FP.”*

Other family planning methods that could be considered but are excluded from the defined modern methods include:

- **Traditional Methods**
- **Periodic Abstinence: Avoiding sexual intercourse during fertile periods (e.g., calendar method, rhythm method).**
- **Withdrawal: The practice of pulling out before ejaculation.**
- **Lactational Amenorrhea Method (LAM)**: Using breastfeeding as a temporary method of contraception.**
- **Natural Family Planning: Techniques that track fertility signs (e.g., basal body temperature, cervical mucus monitoring)**
- **Emergency Contraception: Pills taken after unprotected intercourse to prevent pregnancy (e.g., Plan B, Ella).**
- **Spermicides: Chemical products that kill sperm, often used with barrier methods.**

Including these methods could provide a more comprehensive understanding of family planning options available to women, particularly in different cultural contexts.

*Thank you for this comment. We have included the proportion of women that took non-modern methods of family planning in the results section.*

Based on the findings regarding later uptake of postpartum family planning (FP) among specific groups of women, the following policy recommendations can be made:

- **Targeted Education and Outreach: Develop tailored educational programs aimed at adolescent girls and women with lower levels of education. These programs should focus on the importance of postpartum FP and provide information on available methods, addressing myths and misconceptions.**
- **Strengthening Partner Involvement: Implement initiatives that encourage partner involvement in family planning discussions and decisions. This could include counseling sessions that involve both partners, aiming to promote shared responsibility for contraceptive use.**
- **Enhancing Social Support Networks: Create community-based support systems that provide social and emotional support to postpartum women, particularly those without partners. Peer support groups can help reduce feelings of isolation and improve access to information about FP options.**
- **Financial Assistance Programs: Establish financial support mechanisms for women who lack financial resources from their partners. This could involve subsidies for contraceptive methods or support for accessing healthcare services.**
- **Special Programs for Primigravid Women: Develop specific programs for first-time mothers that address their unique concerns and needs regarding postpartum FP, including counseling on coping with new motherhood and family planning options.**
- **Addressing History of Adverse Pregnancy Outcomes: Provide targeted counseling and support for women with a history of miscarriages or stillbirths, helping them to understand their reproductive health and the benefits of FP.**
- **Integration of FP Services into Maternal Health Care: Ensure that family planning services are integrated into routine maternal and child health visits, particularly for women with young children, to facilitate discussions about postpartum FP.**
- **Ambivalence and Counseling: Offer specialized counseling for women who express ambivalence about their recent pregnancy, helping them to explore their feelings and understand their options for family planning.**

*Thank you for this comment. We have included the suggested policy recommendations in the discussion section.*
