# Supplementary material for: Cofactors of earlier uptake of modern postpartum family planning methods in Kenya
Source: PLOS Glob Public Health. 2025 Jun 6;5(6):e0004353. doi: 10.1371/journal.pgph.0004353 (PMC12143578; doi:10.1371/journal.pgph.0004353)
Supplement: Attachment — Submitted filename: Response letter to reviewers.docx [file pgph.0004353.s006.docx]

**KENYATTA NATIONAL HOSPITAL**	Tel.: 2726300/2726450/2726565

P.O. Box 20723-00202 Nairobi	Research & Programs: Ext. 44705

Fax: 2725272

Email: [knhresearch@gmail.com](mailto:knhresearch@gmail.com)

Date: 4^th^ February 2025

Dear Editor,

We are grateful for the comments on the submission of our manuscript, “*Cofactors of earlier uptake of modern postpartum family planning methods in Kenya”*

We have addressed the comments raised below in italic.

We appreciate your consideration of this manuscript for publication in the PLOS Global Public Health journal. Please contact me if you have any questions regarding this resubmission.

Sincerely,

Author

Comments

1. Results: Consider a more informative caption for figure 1, such as “Cumulative incidence of sexual resumption and postpartum FP uptake in the overall population”

*Thank you for this comment. As advised, we have edited the figure 1 caption in the manuscript to “Cumulative incidence of sexual resumption and postpartum FP uptake in the overall population”.*

*We have also edited figure 4 caption in the manuscript to “Forest plots of cofactors of earlier uptake of modern postpartum family planning methods” reflect the caption in the figure.*
